# CNN-based multi-modal radiomics analysis of pseudo-CT utilization in MRI-only brain stereotactic radiotherapy: a feasibility study

**DOI:** 10.1186/s12885-024-11844-3

**Published:** 2024-01-10

**Authors:** Xin Yang, Bin Feng, Han Yang, Xiaoqi Wang, Huanli Luo, Liyuan Chen, Fu Jin, Ying Wang

**Affiliations:** 1 Departments of Radiation Oncology, Chongqing University Cancer Hospital, No. 181, Han Yu Road, Shapingba District, Chongqing, 400030 People’s Republic of China; 2 Apodibot Medical, Beijing, People’s Republic of China

**Keywords:** Multi-modal radiomics analysis, Pseudo-CT, MRI-only radiotherapy

## Abstract

**Background:**

Pseudo-computed tomography (pCT) quality is a crucial issue in magnetic resonance image (MRI)-only brain stereotactic radiotherapy (SRT), so this study systematically evaluated it from the multi-modal radiomics perspective.

**Methods:**

34 cases (< 30 cm³) were retrospectively included (2021.9-2022.10). For each case, both CT and MRI scans were performed at simulation, and pCT was generated by a convolutional neural network (CNN) from planning MRI. Conformal arc or volumetric modulated arc technique was used to optimize the dose distribution. The SRT dose was compared between pCT and planning CT with dose volume histogram (DVH) metrics and gamma index. Wilcoxon test and Spearman analysis were used to identify key factors associated with dose deviations. Additionally, original image features were extracted for radiomic analysis. Tumor control probability (TCP) and normal tissue complication probability (NTCP) were employed for efficacy evaluation.

**Results:**

There was no significant difference between pCT and planning CT except for radiomics. The mean value of Hounsfield unit of the planning CT was slightly higher than that of pCT. The Gadolinium-based agents in planning MRI could increase DVH metrics deviation slightly. The median local gamma passing rates (1%/1 mm) between planning CTs and pCTs (non-contrast) was 92.6% (range 63.5–99.6%). Also, differences were observed in more than 85% of original radiomic features. The mean absolute deviation in TCP was 0.03%, and the NTCP difference was below 0.02%, except for the normal brain, which had a 0.16% difference. In addition, the number of SRT fractions and lesions, and lesion morphology could influence dose deviation.

**Conclusions:**

This is the first multi-modal radiomics analysis of CNN-based pCT from planning MRI for SRT of small brain lesions, covering dosiomics and radiomics. The findings suggest the potential of pCT in SRT plan design and efficacy prediction, but caution needs to be taken for radiomic analysis.

**Supplementary Information:**

The online version contains supplementary material available at 10.1186/s12885-024-11844-3.

## Background

As a conductor, the brain is composed of gray and white matter, and controls every function and organ of the body. Most of its normal organs, except the spinal cord, are located in the soft tissue and are protected by a hard skull, and any brain tumor that grows in a restricted space can cause serious problems such as blurred or lost vision, epilepsy, speech difficulties [1–3]. Considering its anatomical characteristics, both computed tomography (CT) and magnetic resonance image (MRI) are often used to diagnose intracranial tumors. Meanwhile, CT/MRI-based radiomics also has been used for diagnosis, efficacy assessment, and prediction [4]. As for the treatment of intracranial tumors, surgery is the first choice [5]. When all cancer cells cannot be removed or the tumor location is difficult to reach, radiation therapy (RT) becomes the preferred option [6]. In the past few years, due to screening tests, intracranial tumors have been detected early when it’s small. As for small sizes (≤ 33.5 cm^3^) and numbers (≤ 5) of brain lesions, stereotactic radiotherapy (SRT) has been widely used due to rapid dose fall-off [7–9].

In fact, brain surgeons prefer MRI over CT due to the fact that intracranial tumors are less likely to invade bone, as well as MRI’s advantages such as no radiation damage, high resolution, and excellent soft tissue contrast [10, 11]. Additionally, the rich parameters and sequences, such as T1-Weighted (T1W) and T2-Weighted within MRI, offer oncologists an expanded view of brain structures, significantly enhancing diagnosis and treatment capabilities [12]. Recently MRI-only RT, performing all steps of the RT chain using MRI as the sole modality, was proposed to improve efficiency while avoiding registration errors [13]. However, the intrinsic distortions of MRI may result in geometric inaccuracies in radiotherapy planning [14], and additionally, it lacks pertinent information regarding electron density, which is crucial for dose calculations. To obtain CT-like density information, pseudo-CT (pCT) generated from planning MRI has become a key part of the MRI-only workflow [15].

Nowadays the advancement of deep learning has resulted in the development of numerous methods for generating pCT [16, 17]. To assess the reliability and accuracy of these images, researchers mainly focus on two essential aspects: image segmentation and dose validation. As for image segmentation, the use of objective metrics, such as Hounsfield unit (HU), structural similarity index, and dice similarity coefficient, is prevalent in evaluating the quality and segmentation accuracy of pCT based on convolutional neural network (CNN)[18,19]. In dose validation, both 2D dose-volume histogram (DVH) metrics and 3D gamma passing rates play a critical role in evaluating the deviation of radiation dose delivery [20–22]. Till now, all these studies show significant advantage of CNN-based pCT in both image segmentation and dose validation for lesions with a comparatively larger size [23]. Meanwhile, the combination of MRI and CT in radiomics analysis demonstrates significant advantages over single-modality imaging, and the analysis of CT features is indispensable [24,25]. However, there have been no study that show in detail pCT application in radiomic analysis and efficacy evaluation, especially for brain SRT.

This is the first multi-modal radiomics study to systematically evaluate MRCAT-generated (CNN-based) pCT generated from planning MRI for small intracranial lesions (< 30 cm^3^) with SRT. It addressed the following five questions derived from pCT. (1) What is the difference between pCT and planning CT? (2) What is the dose deviation between them? (3) Are the differences in radiomics features significant? (4) Is there a significant difference in efficacy assessment? (5) What is the correlation among these differences and what factors significantly influence them?

## Methods

### Overall workflow

**Fig. 1:**
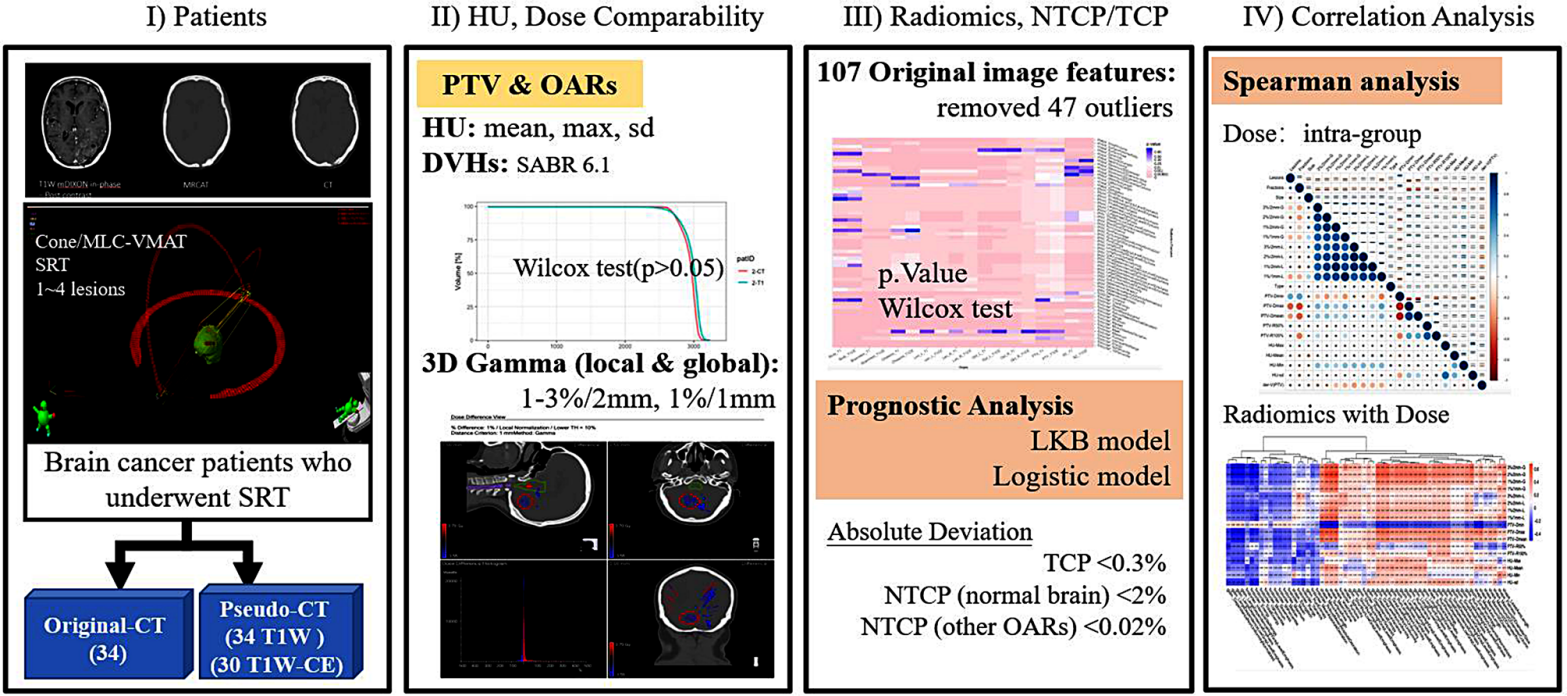
Overall workflow of multi-omics feasibility analysis performed in this study. SRT = stereotactic radiotherapy, T1W = T1-Weighted, T1W-CE = contrast-enhancing T1W, HU = Hounsfield unit, PTV = planning target volume, OARs = organs at risk, DVH = dose-volume histogram, TCP = tumor control probability, NTCP = normal tissue complication probability

The overall workflow of the study is illustrated (Fig. 1). The study consisted of the following steps: (a) acquiring brain patient data from our institution, where the patient underwent SRT, with planning CT as well as planning MRI; (b) comparing the differences in HU and dose between the pCT and initial planning CT, where dose deviation was evaluated using DVH and 3D gamma spatial distribution; (c) comparing the differences in radiomics features and efficacy assessment; (d) analyzing the observed differences and performing correlation analysis to identify the underlying reasons.

### Patient data

Of the forty-one brain cancer patients treated with intra-cranial SRT in our institution between October 2021 to September 2022, thirty-four patients were included in this retrospective study (Table 1). The exclusion criteria for patient selection involved individuals specifically characterized by the presence of implanted metallic objects, cranial deformities, or specific cranial pathologies. Seven patients were excluded from the original dataset due to either metallic artifacts in their scans (n = 3) or image sequence non-compliance (n = 4).

**Table 1:** Patient characteristics

Main Patients Characteristics		*N* = 34 (excluded 7)	%
Gender	Male	17	50.0
	Female	17	50.0
Age median (range)		54(11–74)	
PTV (cm^3^)	Mean (range)	(0.5–27.3)	
	Median	3.4	
	(0,20]	30	32.4
	(20,30)	23	67.6
Number of lesions	1	25	73.5
	2	4	11.7
	3	4	11.7
	4	1	2.9
	Mean Size	1	
Primary Histology	Lung	25	73.5
	Breast	4	11.7
	Brain	2	5.9
	Others	3	8.9
Fraction	1	14	41.2
	3	14	41.2
	5	5	14.7
Type of plan	Cone-VMAT	11	32.4
	MLC-VMAT	23	67.6

*Note*: VMAT = volumetric modulated arc-therapy, MLC = multi-leaf collimator

The study protocol was approved by the Ethics Committee of our institution (Approval No. CZLS2021084-A). Informed consent was obtained from all patients prior to participation in the study. All patient data were de-identified and kept confidential throughout the study.

Each patient had at least one SRT treatment plan with both planning CT and planning MRI acquired as a part of the standard treatment pathway. The number of lesions treated by SRT is less than 5.

### Imaging

Planning CT (Brilliance Big Bore, Philips Medical Systems, Inc., Cleveland, OH) was obtained with a 1 mm slice thickness, a 512 × 512 matrix (pixel size: 1.06 mm×1.06 mm), a tube current of 182 ~ 320 mA, a 120 kV tube voltage. Non-contrast-enhancing agent was used for the planning CT scan.

The planning MRI was acquired on a 3T MRI Unit (Ingenia MR-RT, Philips Medical Systems, Inc., Best, the Netherlands) with the patients in a supine position, and this imaging session occurred on the same day as the planning CT acquisition. The RT head fix devices, comprising of an individually designed foam plastic and thermoplastic mask, in conjunction with coils - a pair of Flex L coils in synergy with a FlexCoverage Posterior Coil and a FlexCoverage Anterior Coil - were meticulously placed on the RT table, known as CouchTop XD. Conventional T1W and T2W scans were followed by a MRCAT Brain protocol, which is a 3D T1W mDIXON MR sequence with scanning duration of 2 min and 51 s, time of repetition 5.5ms, dual times of echo 1.93ms and 3.4ms, and bandwidth 868.3 Hz. The MRI were reconstructed into 400 × 400 × 260 matrix with voxel size of 0.675 × 0.675 × 1.00 mm^3^. Each patient had both T1W and contrast-enhancing T1W (T1W-CE) sequences of MRI (Gadolinium-based contrast agents). The total elapsed time of the MRI scan process for the patient was between 23 and 26 min (including localization, T1W, T2W, and T1W-CE sequence scans).

Automatic rigid registration between the planning CT and planning MRI was performed using Eclipse™ version 15.6 (Varian Medical Systems, Palo Alto, CA, USA).

### Pseudo-CT image generation

The pCT, as called the MRCAT Images, were generated from the MRCAT source images which are the non-contrast-enhancing and contrast-enhancing mDIXON images with the pre-defined MRCAT Brain imaging protocol by Philips RTgo 4.1 post-processing software in the MR console. This post-processing algorithm was performed right after the MRI source images have been reconstructed.

The first step of pCT generation is the pre-processing which ensures the MRCAT source images have consistent scales in regard to the signal intensity, whereas the usual MRI image scaling are not fixed and vary substantially among scan sections depending on scanner post-processing algorithm and receiving coil proximity. Next, the body mask is generated by thresholding the signal intensity, and it is extended according to head-neck morphology. Then the intensity-normalized pair of fat and water 2D images are used as input sequence in the CNN. The CNN had been pre-trained using matched pairs of CT and MRCAT source images acquired with same protocol. The CNN training was performed with the following steps: (a) imaging the same patient using the same positioning device on MRI and CT to ensure good registration; (b) registration of MRI and CT with an initial rigid registration and a deformable registration to improve the match between CT and MRI; (c) CT images are converted from HU-value to mass density using the site-specific calibration tables which were provided along with CT training data; (d) selecting data for development, and training the CNN based on pairs of MRI and mass density; (e) verification performed after the training development using dataset which was kept separate from development dataset. The training of the CNN had been completed before the algorithm was released. Eventually, the 3D pCT volume are then generated from mass density image slices, after re-ordering the 2D slice and converting mass density to HU-values.

With the pCT output from the MRCAT algorithm, validation steps are performed after the CNN, to ensure the pCT images are suitable for radiation treatment. The sanity checks ensure that the imaging field of view has been positioned correctly and that the pCT body outline matches that of the MRI source images. The pCT images are stored into the image database once the post-processing step is finished.

### Treatment planning

Gross tumor volume (GTV) and organs at risk (OARs) were manually delineated by a clinical RT doctor, with the GTV being defined as the macroscopic contrast enhanced lesion from MRI. The planning target volume (PTV) was based on the GTV, with a 2 mm isotropic margin.

The prescribed dose in relation to the PTV varied among different patients, including a uniform dose of 1 × (20–24) Gy, 3 × 7 Gy, and 5 × 6 Gy. For a single lesion in the brain with long axis is less than 17.5 mm, Cone’s non-coplanar arc plan was used. And for multiple lesions in the brain with long axis is more than 17.5 mm, non-coplanar volumetric modulated arc-therapy plan modulated by multi-leaf collimator was used [9]. To ensure a rapid reduction in the external dose to the PTV and minimize the dose to OARs, the maximum dose is limited to the PTV, with the prescribed dose maintained at approximately 80% of the maximum dose. The prescription, dose constraints, and optimization objectives were all adhered to in compliance with the UK version 6.1 of the Stereotactic Ablative Radiotherapy guideline [26].

### Dose calculations

For the planning CT, the dose calculation grid was set to 1.25 × 1.25 × 1.25 mm^3^. Eclipse™ version 15.6 allowed us to copy the original CT’s plan to pseudo-CT, and recalculate the dose using same initial radiotherapy plan (with the corresponding isocenter, beam configuration, MU, etc.) without re-optimization.

The premise of using the plan of planning CT is to replicate the delineation of PTV and OARs. And the doctor’s original delineation information is stored on the MRI. In order to reduce the registration error of the spatial coordinates, the rigid registration relationship of the original CT-MRI is used between the pCT and the planning CT.

For dose comparison purpose, the pCT’s dose map was transferred to the original CT using “Dose Accumulation-Rigid” tool in MIM version 6.6 (MIM® software Inc., Cleveland, OH, USA). In this tool, instead of accumulated dose map, we export rigid dose map of pCT’s plan. Since the rigid registration relationship on the original TPS can’t be used here, the subsequent gamma analysis has a certain error effect.

### HU-comparability

For HU value comparison purpose, we counted the maximum, minimum, mean and standard deviation of HU-value for OARs (include brain, brainstem, lens, optic chiasm, optic nerve, and spinal cord) and PTV of all images.

### Dose-volume histogram

DVHs were extracted from each initial and synthetic dose map for PTVs and OARs, following the UK version 6.1 of the Stereotactic Ablative Body Radiation Therapy guideline [26]. Maximum dose (D_max_, D_0.1 cm³_) were collected for optic chiasm, optic nerves, lens, brainstem, and spinal cord. D_50%_ (D_x%_ = dose received by x% of the volume) of normal brain were collected. Regarding the PTVs, the Radiation Therapy Oncology Group uses R_100%_ and R_50%_ instead of Conformity Index and gradient index to represent high volume overflow and gradient indices [26]. Vol (100%) and Vol (50%) are the volumes of the patient receiving at least 100% and at least 50% of the prescription dose respectively. Since Vol (100%) is sensitive to target volume deficit, and the UK version 6.1 of the Stereotactic Ablative Body Radiation Therapy guideline has slightly modified R_100%_ and R_50%_, R_100%_= Vol (100%)/Vol (PTV), R_50%_=Vol (50%)/Vol (PTV). And the guideline mentioned that 95% of PTV receives at least 100% of the quoted prescribed dose.

To better compare the changes in dose due to image differences of PTVs, we normalized the all plans’ D_95%_ of PTVs received 100% of prescribed dose. Therefore, D_min_, D_max_, D_mean_, R_50%_, R_100%_ of PTVs were measured. Since prescribed dose varied among patients, comparisons of Dmin, Dmax, and Dmean metrics for PTV were normalized by dividing by prescribed dose. Meanwhile, the low-dose OARs were not standardized, and only the 30 patients with T1W-CE sequences were included in the comparison of the two groups of pCTs.

### Gamma analysis

For dose comparison purposes, the synthetic dose map was then rigidly aligned to the planning CT using MIM version 6.6 (MIM® software Inc., Cleveland, OH, USA). The 3D gamma analysis (1–3%/2 mm, 1%/1 mm) was conducted using 3DVH (Sun Nuclear Corporation, Melbourne, FL, USA), with a 10% threshold to the maximum dose [27]. The distribution of cold and hot spots in the dose of one patient in the study, with a local 3D dose deviation standard of 1%/1 mm, was illustrated in the Supplemental Fig. E1. It should be noted that the rigid alignment of MIM in this process may introduce error, which could potentially bias the final gamma comparison results.

### Radiomics features

Only the original image features were extracted. The differences in radiomics features of PTVs and OARs were compared separately. PyRadiomics package (python3.7) is used to accomplish this operation. The radiomics features contain the following 7 categories: first order statistics, 2D/3D shape-based, gray level cooccurrence matrix, gray level run length matrix, gray level size zone matrix, neighboring gray tone difference matrix, and gray level dependence matrix. When analyzing the differences, some outliers were removed, and these outliers may have been caused by a maximum or minimum value presented after extraction because the organ was too small.

### Prognostic analysis

To evaluate the clinical effects of different images, quantitative biological indices including tumor control probability (TCP) of PTV and normal tissue complication probability (NTCP) of OARs were calculated using the python program. TCP/NTCP assessment belongs to the Radiation Radiobiology Radiomics, utilizing the characteristics of patients and tumors to predict the efficacy and side effects of RT. TCP was computed by Logistic model which based on Equivalent Uniform Dose model [28], and NTCP was computed based on Lyman-Kutcher-Burman model [29].

In scenarios where the biological equivalent dose is 2 Gy, the parameters for the computational models of TCP and NTCP employed in this study can be found in the Supplemental Table E1. Where $$ \alpha /\beta $$ is the tissue-specific linear-quadratic parameter for the exposed organ, $$ {TCD}_{50}$$ is the tumor dose to control 50% of the tumor when the tumor is homogeneously irradiated, $$ {\gamma }_{50}$$ is the change in TCP expected because of a 1% change in dose about the $$ {TCD}_{50}$$,$${TD}_{50}$$ is the uniform dose given to the entire organ volume that results in 50% complication risk, *m* is a measure of the slope of the sigmoid curve represented by the integral of the normal distribution, *n* is a parameter that describes the magnitude of the volume effect [30, 31].

### Statistical analysis

In the significance analysis, since not all statistics follow a normal distribution (α = 0.05), the Wilcoxon test [32] was used to compare the planning CTs and pCTs. The significant difference level was set at 0.05. And the Spearman correlation analysis [33] was used to analyze the correlates of dose deviation. Statistical analyses were performed with R (version 4.2.2).

## Results

### HU comparability

Except for the small-volume lenses (both left and right) with a volume < 0.2 cm³, the average HU deviation for the OARs and PTV consistently remains below 15 HU, as indicated in Supplemental Table E2. And the box distribution of HU-max, HU-mean, HU-min, HU-SD, and volume deviation values between planning CT and pCT was provided in the Supplemental Fig. E2. Then we found that the HU-mean of the planning CT was higher than that of the pCT.

### DVH comparisons

Analysis of OARs and PTV indicated no significant difference among all studied OARs and PTV, with p-values much greater than 0.05 (Table 2). But the dose values for PTV and OARs (excluding the brainstem) in the pCT were lower than those in the initial CT.

**Table 2:** DVH comparisons between the Initial dose and the synthetic dose maps for OARs and PTV

DVH Feature		Initial CT		T1W	T1W-CE		MAD			P value	
		*N* = 34	*N* = 30	*N* = 34		*N* = 30	①	②	③	T1W	T1W-CE
PTV	D_min_(Gy) / PD	0.85	0.84	0.84		0.84	0.01	0.00	0.00	0.538	0.523
	D_max_(Gy) / PD	1.18	1.18	1.18		1.18	0.00	0.00	0.00	0.995	0.995
	D_mean_(Gy) / PD	1.09	1.09	1.09		1.09	0.00	0.00	0.00	0.879	0.879
	R_50%_	4.29	4.25	4.09		4.07	0.20	0.18	0.02	0.536	0.536
	R_100%_	1.31	1.32	1.26		1.25	0.05	0.07	0.01	0.519	0.519
D_max_(Gy) 0.1 cm³	Lens_L	22.63	15.16	21.84		13.67	0.79	1.49	-0.05	0.621	0.621
	Lens_R	18.04	14.62	17.23		14.10	0.81	0.52	0.02	0.950	0.950
	Opt_Chiasm	69.52	61.27	63.40		52.14	6.12	9.13	-0.05	0.648	0.648
	Opt_Nerve_L	73.83	63.41	73.68		64.48	0.15	-1.07	-0.05	0.956	0.956
	Opt_Nerve_R	54.82	43.59	57.04		45.31	-2.22	-1.72	0.11	0.931	0.961
	Spinal Cord	52.45	44.28	52.19		42.87	0.26	1.41	-0.12	0.893	0.893
	Brainstem	353.54	358.28	355.23		359.15	-1.69	-0.87	-0.21	0.971	0.971
D50%(Gy)	Brain-GTV	76.94	68.48	78.26		69.37	-1.32	-0.89	-0.05	0.917	0.917

*Note*: DVH features are expressed as mean values, and the shaded region in the MAD comparison plot represents data from only 30 patients. ① = CT vs. T1W; ② = CT vs. T1W-CE; ③ = T1W vs. T1W-CE. PD = prescribed dose; Lens_L = left lens; Lens_R = right lens; Opt_Chiasm = optic chiasm; Opt_Nerve_L = left optic nerve; Opt_Nerve_R = right optic nerve; Brain-GTV = normal brain

The Mean Absolute Deviation (MAD) data from the two groups of pCTs indicated that the use of contrast agents had no significant effect on the dose deviation of the generated pCTs. However, in comparing the PTV and OARs (excluding the right lens and right optic nerves), it was observed that the dose data of the pCT generated by the T1W sequence were marginally superior to those generated by the T1W-CE sequence. And the small size and irregular structure of the optic chiasm, compared to other organs, slightly increased the MAD of pCT.

### Local and global gamma analysis

The median 3D local gamma passing rates (1%/2 mm, 1%/1 mm) between planning CTs and pCTs (T1W) were 99.4% (range 88.1-100%), and 92.6% (range 63.5-99.6%), respectively. Moreover, the 3D dose deviation of the pCT generated by non-contrast-enhancing and contrast-enhancing MRI was less than 1% for any criterion.

**Fig. 2:**
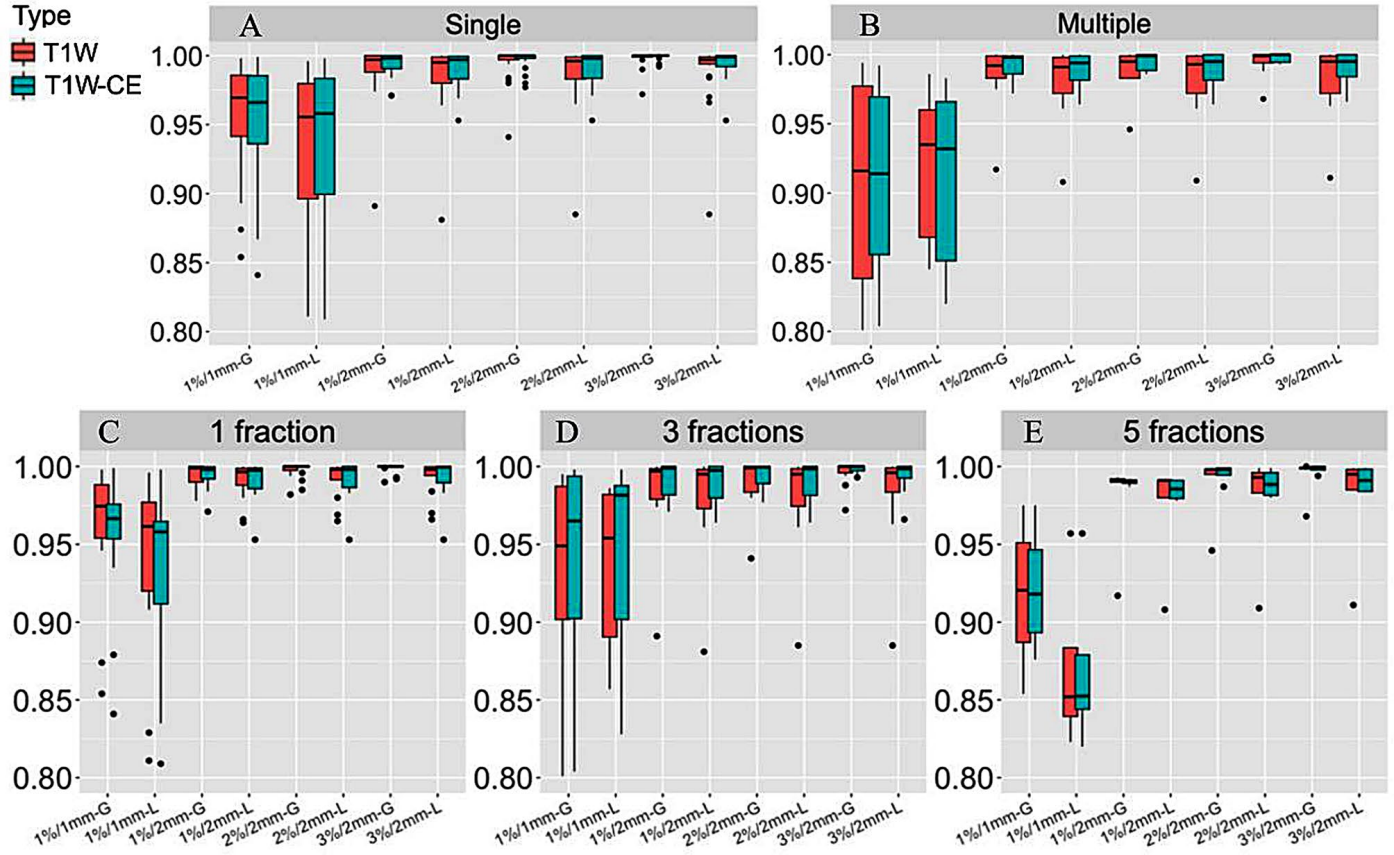
The box distribution of 3D gamma passing rates. (**A**) single lesion; (**B**) multiple lesions; (**C**) 1 fraction SRT; (**D**) 3 fractions SRT; (**E**) 5 fractions SRT. “-G” = global gamma, “-L” = Local gamma

Box plots were used to show the gamma passage rate under different numbers of lesions and fractions (Fig. 2), while the mean values for each group were provided in the Supplemental Table E3-E4. Our findings indicate that the passing rate of global gamma analysis gradually decreases with stricter criteria, while the passing rate of local gamma analysis shows a significant decrease between 2 mm and 1 mm criteria. Moreover, the number of lesions and fractions can influence the deviation in 3D dose distribution.

Analysis for the 1%/1 mm local gamma results revealed that among these patients: pass rate > 80%, D_min_ bias was almost absent, and D_max_ bias may exist; however, pass rate ≤ 80%, both D_min_ and D_max_ bias were significant. Some example diagrams of DVH were provided in the Supplemental Fig. E3. But for a single lesion, there is only one case where the numerical deviation occurred, with a gamma pass rate of 1% for 1 mm falling below 80%. We further found its morphologic configuration has been close to 2 lesions in the Supplemental Fig. E4.

### Radiomics features difference

A total of 107 original image features were extracted for OARs and PTVs. Of these, 47 outliers were removed, which contained all ngtdm and glcm features (showed in the Supplemental Table E5). For these 60 features, the p values between the pCTs generated by non-contrast-enhancing and contrast-enhancing MRI were both greater than 0.95, and there was no difference between them.

Comparing between pCTs generated by T1W-CE MRI and the planning CTs, the p-value heat map results for PTVs and OARs are shown in the Supplemental Fig. E5. And we found features with p-values greater than 0.05: ten features for PTV, nine features for spinal cord, two features for brainstem, and none for other organs. This indicates that there are significant differences in overall radiomic features between the CNN-based pCT and the planning CT. Therefore, the pCT generated by AI cannot be considered as a substitute for the planning CT in radiomic analysis.

### Prognostic analysis

The parameters (showed in the Supplemental Table E1) and differential DVH [31] of these 3 type images are used to calculate TCP and NTCP. And we calculated the mean, range and absolute deviation of TCP/NTCP for PTV and OARs (Table 3). One of the patients had a lesion located in the brainstem, and the results of the NTCP evaluation of his brainstem are listed separately.

**Table 3:** Mean values of TCP/NTCP of OARs and PTV

TCP/NTCP (Mean, range)	Initial (34)	T1W	T1W-CE	Absolute Deviation (T1W)	Absolute Deviation (T1W-CE)
PTV	99.58% (99.19–99.87%)	99.59% (99.18–99.87%)	99.60% (99.18–99.87%)	0.03% (0.00-0.22%)	0.03% (0.00-0.22%)
Lens	0.13‰ (0.11–0.24‰)	0.13‰ (0.11–0.26‰)	0.13‰ (0.11–0.24‰)	0.00‰ (-0.03-0.01‰)	0.00‰ (-0.01-0.01‰)
Opt_Chiasm	2.36‰ (2.14–4.67‰)	2.35‰ (2.14–4.58‰)	2.34‰ (2.14–4.59‰)	0.00‰ (-0.07-0.17‰)	0.00‰ (-0.08-0.08‰)
Opt_Nerve	2.23‰ (2.14–2.85‰)	2.23‰ (2.14–2.88‰)	2.22‰ (2.14–2.88‰)	0.00‰ (-0.04-0.03‰)	0.00‰ (-0.04-0.01‰)
Brainstem (33)	0.02‰ (0.00-0.65‰)	0.02‰ (0.00-0.56‰)	0.02‰ (0.00-0.57‰)	0.00‰ (0.00-0.09‰)	0.00‰ (0.00-0.08‰)
Brainstem (PTV)	96.87%	97.36%	97.37%	-0.48%	-0.50%
Brain-GTV	10.06‰ (0.00-49.74‰)	9.12‰ (0.00-46.04‰)	10.12‰ (0.00-44.95‰)	0.94‰ (-4.14-18.12‰)	1.18‰ (-4.14-16.33‰)

*Note*: TCP and NTCP values are expressed as mean values, with a range of values in parentheses; and 0.00‰ in the table is not 0, but represent the value is less than $1{\times 10}^{-6}$. Brainstem (33) = brainstem in 33 patients (PTV outside the brainstem); Brainstem (PTV) = brainstem (the only case of PTV in the brainstem); Opt_Chiasm = optic chiasm; Opt_Nerve = optic nerve; Brain-GTV = normal brain

Our findings showed that the MAD in TCP was 0.03%, while the NTCP of normal brain was 1.06‰, with NTCP values of other OARs below $1{\times 10}^{-6}$. Furthermore, all differences in TCP were below 0.3%, the NTCP values of normal brain were below 2%, the NTCP values of other OARs were below 1‰. These results illustrate the extremely small difference between pCT and planning CT in the efficacy assessment of TCP/NTCP after its use for dose calculation. However, it is worth noting that the deviation in normal brain was slightly larger than that observed in other organs. Additionally, the patient, whose PTV was located within the brainstem, exhibited a significant NTCP bias in the same region.

### Correlation analysis

**Fig. 3:**
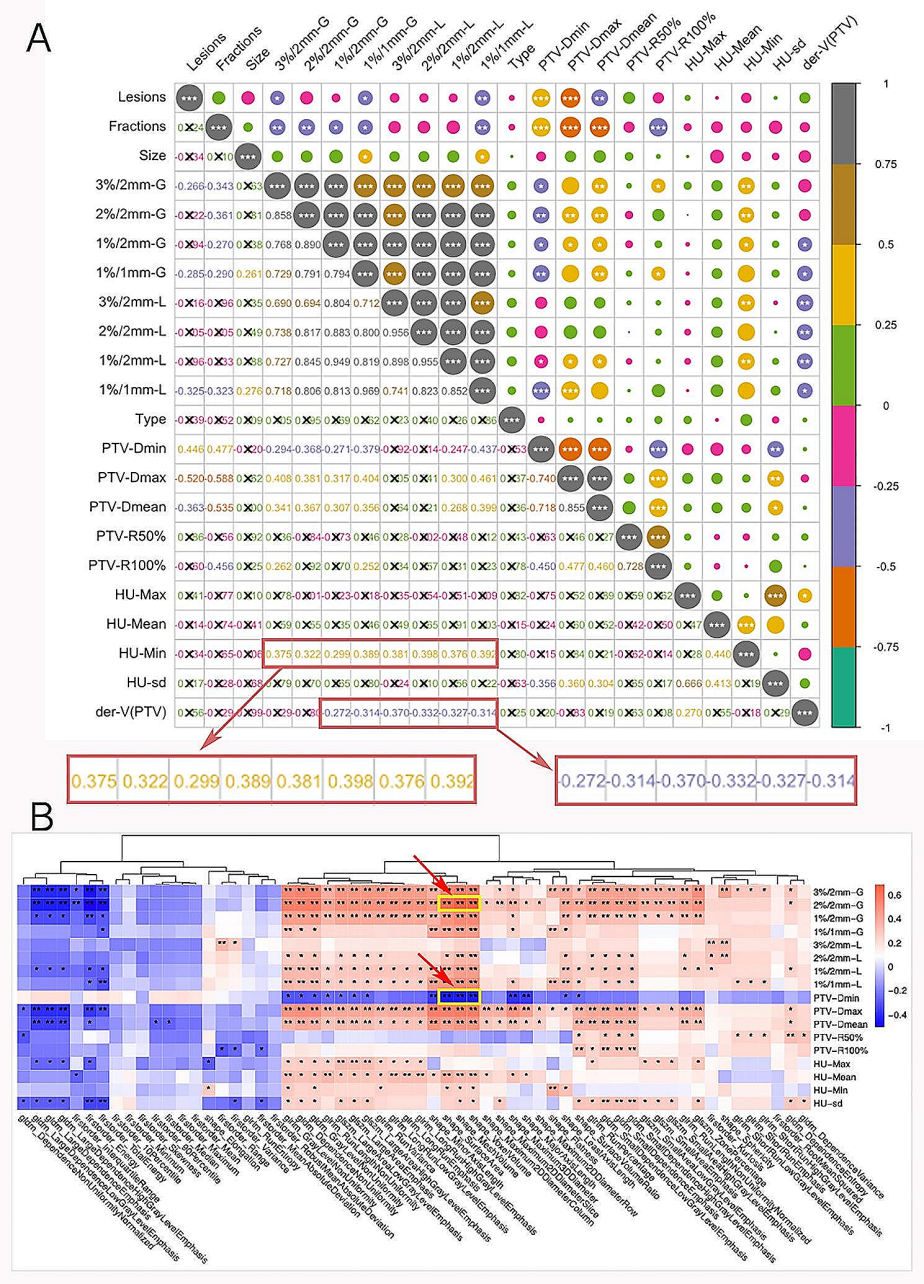
Correlation analysis map: (A) inter-group analysis of factors associated with dose deviation of PTV; (B) intra-group between factors related to dose deviation and radiomic features of PTV. The correlation coefficient matrix values range from − 1 to 1, representing a perfectly negative and positive correlation, respectively. The values close to 0, the smaller the correlation. The color, value, and circle size in the figure indicate the value of the correlation coefficient. “*” is used as the Wilcoxon-test significance label, < 0.05 is indicated by “*”, < 0.01 is indicated by “**”, and < 0.001 is indicated by “***”. And “ × ” indicates that the result did not pass the significance test. “-G” = global gamma, “-L” = Local gamma, der-V(PTV) = PTV volume deviation between pCT and planned CT, Lesions = number of lesions, Fractions = number of fractions, Type = planning MRI sequence (T1W or T1W-CE)

This study involved both inter-group and intra-group Spearman correlation analysis, and the results of the correlation analysis were showed (Fig. 3).

Inter-group correlation analysis included the factors of number of lesions, number of SRT fractions, PTV size, 3D gamma passing rates, type of planning MRI (T1W or T1W-CE), DVH metrics of PTV, HU metrics of PTV, and volume deviation of PTV. We found a higher correlation between the number of lesions and the DVH index, and a correlation between the number of fractions and 2D/3D dosimetric parameters (Fig. 3A). However, PTV size is less associated with them all. It is worth noting that HU-min and PTV volume deviation correlate with 3D dose distribution. The volume deviation is caused by image alignment and differences in the number of layers and layer thickness. And Confidence intervals for intra-group correlations are shown in the Supplemental Fig. E6.

In the radiomic analysis, we found significant differences in most of radiomic features. Further, intra-group correlation analysis was performed to investigate whether these feature differences were associated with dose bias. A strong correlation between three radiomic features, namely Shape Voxel Volume, Shape Mesh Volume, and Shape Surface Area, with the dose distribution, was demonstrated (Fig. 3B). These features represent spatial and shape information, further corroborating that differences in shape variation due to alignment have the most prominent effect on dose distribution. And values of inter-group correlation coefficients are demonstrated in the Supplemental Fig. E7.

## Discussion

The clinical feasibility of CNN-based pCT for small intracranial lesions (< 30 cm^3^) in SRT has not been validated. This study represents the first comprehensive multi-modal radiomics analysis evaluating the feasibility of pCT based on planning MRI. And the study encompasses assessments various domains including image quality, dose validation, radiomic features, and treatment efficacy evaluation, involving statistics, dosimetry, radiomics, and dose-response radiomics. In this study, we found there were no significant differences between pCT and planning CT in these aspects, except for radiomics.

Compared to other studies on pCT feasibility analysis of the brain, this study achieves similar results in terms of HU differences in soft tissue[19, 23, 34]. In this study, the bone MAD was < 30 HU, and the root mean square error was < 113 HU; for soft tissue, the MAD was < 30 HU, and the root mean square error was < 12 HU. Although we successfully demonstrate HU performance in the spinal cord, the HU comparison in the entire cranial skeleton is somewhat unbalanced due to our study specific focus on small brain lesions. Regarding the issue that HU-mean of planning CT was higher than that of pCT, various factors, including patient positioning, scanning parameters such as voltage and current, and image reconstruction techniques, can significantly impact HU values [35]. However, these biases can be mitigated through the application of an electron density table conversion. The observed discrepancies can be largely attributed to the differences in calibration of the electron density Table [36]. Moreover, during the pCT generation process, the low-energy signal within the resultant image is smoothed, which is the underlying cause of the HU-min bias. This has an impact on the dose deviation.

Regarding dose deviations, the pCT generated in this study outperformed other diagnostic MRI-based pCTs, meeting the same evaluation criteria[23, 34], but the use of contrast agents, fractionation of treatment, and the number and morphology of lesions can impact it. Firstly, the pCT derived from the T1W sequence outperformed the T1W-CE sequence (Gadolinium-based agents). This superiority largely stems from a more abundant T1W MRI dataset in the training set. Sample size greatly influences the network’s generative capacity [37]. Additionally, due to the copying and subsequent recalculation of the dose from planning CT onto the pseudo-CT, larger deviations are observed in smaller organs, especially in Dmax. This is attributed to various factors, including differences in structure voxels and inaccuracies in CT-MR/pCT registration. Meanwhile, the 3D gamma analysis revealed an inverse relationship: as the number of fractions and lesions increased, the pass rate correspondingly decreased. The influence of the fraction number is logical, as the restriction criteria vary among fractions, with the threshold escalating with an increase in the number of fractions [26]. And multiple targets lead to heightened plan complexity, tissue deformation, and dose stacking. As the number of targets increases, a corresponding decrease in dose deviation is also observed during dose validation [38].

As for the feasibility analysis of radiomics, the size of organ structures influences radiomic features such as spatial resolution [39]. In our study, the small tumor lesions in the brain primarily involved PTVs and OARs with relatively smaller volumes, which led to certain limitations in our image analysis. Meanwhile, we also observed a higher correlation between shape-related features and dose deviation. The shape variation of the organ in this study arises from layer thickness difference, layer number difference, and alignment. Previous studies have reported CT-MRI registration can lead to significant volumetric errors of between 2 and 3 mm for different sites, causing dose deviation, especially for small structures or targets close to sensitive organs [40]. This further emphasizes the significant impact of registration-induced structural deformation on dose deviation and highlights the necessity of implementing MRI-Only approaches. Therefore, the results indicate that pCT cannot replace the planning CT with small lesions in the brain, and further confirm the impact of image registration on dose deviation from a radiomics perspective. Although, variations in image acquisition parameters can impact the results of radiomics analysis, post-processing standardization could be employed to calibrate and correct the images, enabling the utilization of pseudo-CT images in various radiomics analyses [41].

And the minimal disparity in TCP/NTCP highlights the viability of pCT for assessing patient outcomes in a clinical context. Since the calculation of TCP/NTCP originates from the DVH, a small difference in DVH results inevitably leads to a relatively better outcome in terms of TCP/NTCP. As for the computational model parameters of TCP/NTCP, we excluded spinal cord because due to Lyman-Kutcher-Burman model does not accurately predict its tolerance to SRT [42]. Except for the updated optic pathway parameters of SRT [43], the parameters proposed by Burman et al. were still used for the OAR, with the $\alpha /\beta$ ratio for normal tissues is 3 Gy, whereas the TCP parameters were used for the 1-year local control [8]. Based on the fact that the brainstem NTCP was offset in patients whose PTV was located in the brainstem, it was hypothesized that the accuracy of pCT for PTV delineation within the brainstem region might be slightly reduced. Nonetheless, this presumption necessitates further validation through an extended sample size.

However, this study faces several critical limitations associated with the pCT image generation technique. Firstly, the minor dose discrepancy may be attributable to the small sizes of brain lesions treated with SRT, resulting in minimal radiation exposure to OARs. The findings’ applicability to larger brain tumors may be limited in the scope of this study. Secondly, despite yielding rich results, the study grapples with a limited sample size, emphasizing the need for further expansion to enhance statistical robustness. Additionally, the study only explores the image generation performance of a packaged AI-based pCT generation tool, without investigating the differences in pCT generation using different AI techniques. Furthermore, the study lacks a prospective design and does not employ a pCT for treatment planning but instead replicates plans from the planning CT for analysis. It does not fully demonstrate the real-world application of pCT in clinical settings. It is essential to underscore that non-standard anatomical structures and skull deformities may impact the usability of this Pseudo-CT, and the use of contrast agents may introduce biases in its generation. Special attention should be given to the inapplicability of this technique to patients with implanted metal devices. To enhance the reliability and generalizability of the study, future research can consider increasing the sample size, comparing the differences among various AI-generated methods, and employing a prospective study design to validate the findings of this study.

## Conclusions

Our study comprehensively compares the feasibility of CNN-based pCT derived from planning MRI in SRT of small brain lesions (< 30 cm^3^) using a multi-modal radiomics approach encompassing dosimetry and radiomics. The results demonstrate pCT and planning CT showed similar performance in terms of RT plan design and TCP/NTCP evaluation. However, caution should be exercised when using pCT for imaging analysis and diagnosis.

### Electronic supplementary material

Below is the link to the electronic supplementary material.

Supplementary Material 1

## Data Availability

The data that support the findings of this study are available from the corresponding author, upon reasonable request.

## Abbreviations

pCT: pseudo-computed tomography
MRI: magnetic resonance image
CT: computed tomography
CNN: convolutional neural network
SRT: stereotactic radiotherapy
DVH: dose volume histogram
TCP: tumor control probability
NTCP: normal tissue complication probability
RT: radiation therapy
T1W: T1-Weighted
T1W-CE: contrast-enhancing T1W
HU: Hounsfield unit
MAD: mean absolute deviation
PTV: planning target volume
GTV: gross tumor volume
OARs: organs at risk
