# Supplementary material for: CNN-based multi-modal radiomics analysis of pseudo-CT utilization in MRI-only brain stereotactic radiotherapy: a feasibility study
Source: BMC Cancer. 2024 Jan 10;24:59. doi: 10.1186/s12885-024-11844-3 (PMC10782704; doi:10.1186/s12885-024-11844-3)
Supplement: Supplementary file 1 — Supplementary Material 1 [file 12885_2024_11844_MOESM1_ESM.docx]

**Supplementary Material 1.**

**Table E1: Model Parameters in the TCP and NTCP**

| $\alpha/\beta(Gy)$ | Organ | m/$\gamma_{50}$ | n | ${TD}_{50}$/${TCD}_{50}$ (Gy) |
| --- | --- | --- | --- | --- |
| 1.6 | Optic Chiasma/Optic Nerves | 0.35304 | 0.25 | 157.3 |
| 3 | Lens | 0.27 | 0.3 | 18 |
| 3 | Normal Brain (Brain-GTV) | 0.15 | 0.25 | 60 |
| 3 | Brain Stem | 0.16 | 0.14 | 60 |
| 20 | PTV size:(0,20] cc | 12.44 | - | 0.7617 |
| 20 | PTV size:(20,40] cc | 11.21 | - | 0.9749 |

**Table E2: Mean Value of the Difference in HU Values between Pseudo-CTs and Planning CTs for OARs and PTV**

| Type | Bone | Brain | Brain Stem | Left Len | Right Len | | Optic Chiasma | | Left Optic Nerve | Right Optic Nerve | Spinal Cord | PTV |
| --- | --- | --- | --- | --- | --- | --- | --- | --- | --- | --- | --- | --- |
| T1w | 29.49 | 8.76 | 3.70 | 28.13 | | 23.11 | | -0.57 | 9.01 | 13.05 | -1.29 | 8.11 |
| T1w-ce | 21.5 | 10.00 | 4.48 | 30.50 | | 25.38 | | 2.45 | 9.34 | 7.83 | 1.36 | 8.42 |

**Table E3: Mean Values of 3D Gamma Pass Rates for Single and Multiple Lesions**

| MRI sequence | Number of lesions | Global | | | | Local | | | |
| --- | --- | --- | --- | --- | --- | --- | --- | --- | --- |
|  |  | 3%/2mm | 2%/2mm | 1%/2mm | 1%/1mm | 3%/2mm | 2%/2mm | 1%/2mm | 1%/1mm |
| T1w | 1 | 99.81% | 99.46% | 99.89% | 94.37% | 98.97% | 98.76% | 98.57% | 91.87% |
|  | 2-4 | 99.38% | 98.87% | 98.31% | 88.91% | 97.92% | 97.82% | 97.76% | 82.12% |
| T1w-CE | 1 | 99.90% | 99.66% | 99.28% | 91.14% | 99.39% | 99.17% | 99.01% | 93.30% |
|  | 2-3 | 99.74% | 99.46% | 99.14% | 90.86% | 98.99% | 98.89% | 98.84% | 85.39% |

**Table E4: Mean Values of 3D Gamma Pass Rates for Single and Multiple Factions**

| MRI sequence | Number of Fractions | Global | | | | Local | | | |
| --- | --- | --- | --- | --- | --- | --- | --- | --- | --- |
|  |  | 3%/2mm | 2%/2mm | 1%/2mm | 1%/1mm | 3%/2mm | 2%/2mm | 1%/2mm | 1%/1mm |
| T1w | 1 | 99.92% | 99.76% | 99.42% | 96.05% | 99.29% | 99.19% | 99.06% | 94.04% |
|  | 3 | 99.62% | 99.07% | 98.45% | 91.48% | 99.45% | 98.18% | 98.06% | 86.91% |
|  | 5 | 99.30% | 98.72% | 97.66% | 88.50% | 97.76% | 97.60% | 97.24% | 83.12% |
| T1w-CE | 1 | 99.89% | 99.80% | 99.42% | 95.44% | 99.27% | 99.20% | 99.13% | 93.64% |
|  | 3 | 99.83% | 99.43% | 99.12% | 85.61% | 99.38% | 99.05% | 98.95% | 90.38% |
|  | 5 | 99.83% | 99.55% | 99.00% | 92.18% | 99.13% | 98.90% | 98.50% | 87.05% |

**Table E5: 40 Excluded Radiomics Feature Names**

| Feature-Brain-40 | T1w | T1w-CE |
| --- | --- | --- |
| firstorder_Entropy | 3.79E-15 | 7.11E-15 |
| firstorder_Uniformity | 3.79E-15 | 7.11E-15 |
| glcm_Autocorrelation | 3.08E-15 | 7.11E-15 |
| glcm_ClusterProminence | 1.04E-15 | 4.33E-15 |
| glcm_ClusterShade | 1.30E-15 | 5.56E-15 |
| glcm_ClusterTendency | 2.49E-15 | 5.56E-15 |
| glcm_Contrast | 5.91E-08 | 2.76E-07 |
| glcm_Correlation | 7.31E-13 | 9.34E-12 |
| glcm_DifferenceAverage | 5.91E-08 | 2.76E-07 |
| glcm_DifferenceEntropy | 5.91E-08 | 2.76E-07 |
| glcm_DifferenceVariance | 5.91E-08 | 2.76E-07 |
| glcm_Id | 5.91E-08 | 2.76E-07 |
| glcm_Idm | 5.91E-08 | 2.76E-07 |
| glcm_Idmn | 5.91E-08 | 2.76E-07 |
| glcm_Idn | 5.91E-08 | 2.76E-07 |
| glcm_Imc1 | 2.20E-09 | 7.28E-09 |
| glcm_Imc2 | 6.53E-16 | 6.27E-16 |
| glcm_InverseVariance | 5.91E-08 | 2.76E-07 |
| glcm_JointAverage | 1.03E-14 | 1.82E-14 |
| glcm_JointEnergy | 3.79E-14 | 2.19E-13 |
| glcm_JointEntropy | 9.08E-14 | 5.55E-13 |
| glcm_MCC | 7.31E-13 | 9.34E-12 |
| glcm_MaximumProbability | 3.79E-14 | 1.81E-13 |
| glcm_SumAverage | 1.03E-14 | 1.82E-14 |
| glcm_SumEntropy | 5.41E-14 | 2.65E-13 |
| glcm_SumSquares | 1.03E-14 | 1.82E-14 |
| gldm_GrayLevelVariance | 3.79E-15 | 7.11E-15 |
| gldm_HighGrayLevelEmphasis | 3.79E-15 | 7.11E-15 |
| gldm_LowGrayLevelEmphasis | 3.79E-15 | 7.11E-15 |
| glrlm_GrayLevelNonUniformityNormalized | 8.25E-16 | 2.84E-14 |
| glrlm_GrayLevelVariance | 8.25E-16 | 2.84E-14 |
| glrlm_HighGrayLevelRunEmphasis | 8.25E-16 | 3.53E-14 |
| glrlm_LowGrayLevelRunEmphasis | 8.25E-16 | 3.53E-14 |
| glszm_GrayLevelNonUniformity | 0.63676382 | 0.19419342 |
| glszm_GrayLevelNonUniformityNormalized | 0.00434693 | 0.00051839 |
| glszm_GrayLevelVariance | 0.00434693 | 0.00051839 |
| glszm_HighGrayLevelZoneEmphasis | 0.00434693 | 0.00051839 |
| glszm_LowGrayLevelZoneEmphasis | 0.00434693 | 0.00051839 |
| glszm_SizeZoneNonUniformity | 0.95627593 | 0.64474825 |
| glszm_SizeZoneNonUniformityNormalized | 0.82164823 | 0.80479058 |
| glszm_ZoneEntropy | 0.87894618 | 0.8673846 |
| glszm_ZoneVariance | 8.15E-06 | 0.00010973 |
| ngtdm_Busyness | 8.50E-13 | 8.01E-12 |
| ngtdm_Coarseness | 3.96E-10 | 1.27E-11 |
| ngtdm_Complexity | 5.42E-08 | 1.93E-07 |
| ngtdm_Contrast | 3.15E-12 | 1.89E-09 |
| ngtdm_Strength | 1.15E-12 | 1.81E-09 |

Note.—These features were extracted with the same values in both OARs and PTVs. These features of body were extracted and significance analysis was done with pCT to analyze the reason for this phenomenon.

All values were found to be significantly different except for glszm_ZoneEntropy, glszm_SizeZoneNonUniformityNormalized, and glszm_SizeZoneNonUniformity. It indicates that all features except these three non-differential features could not be extracted due to organ size limitation.


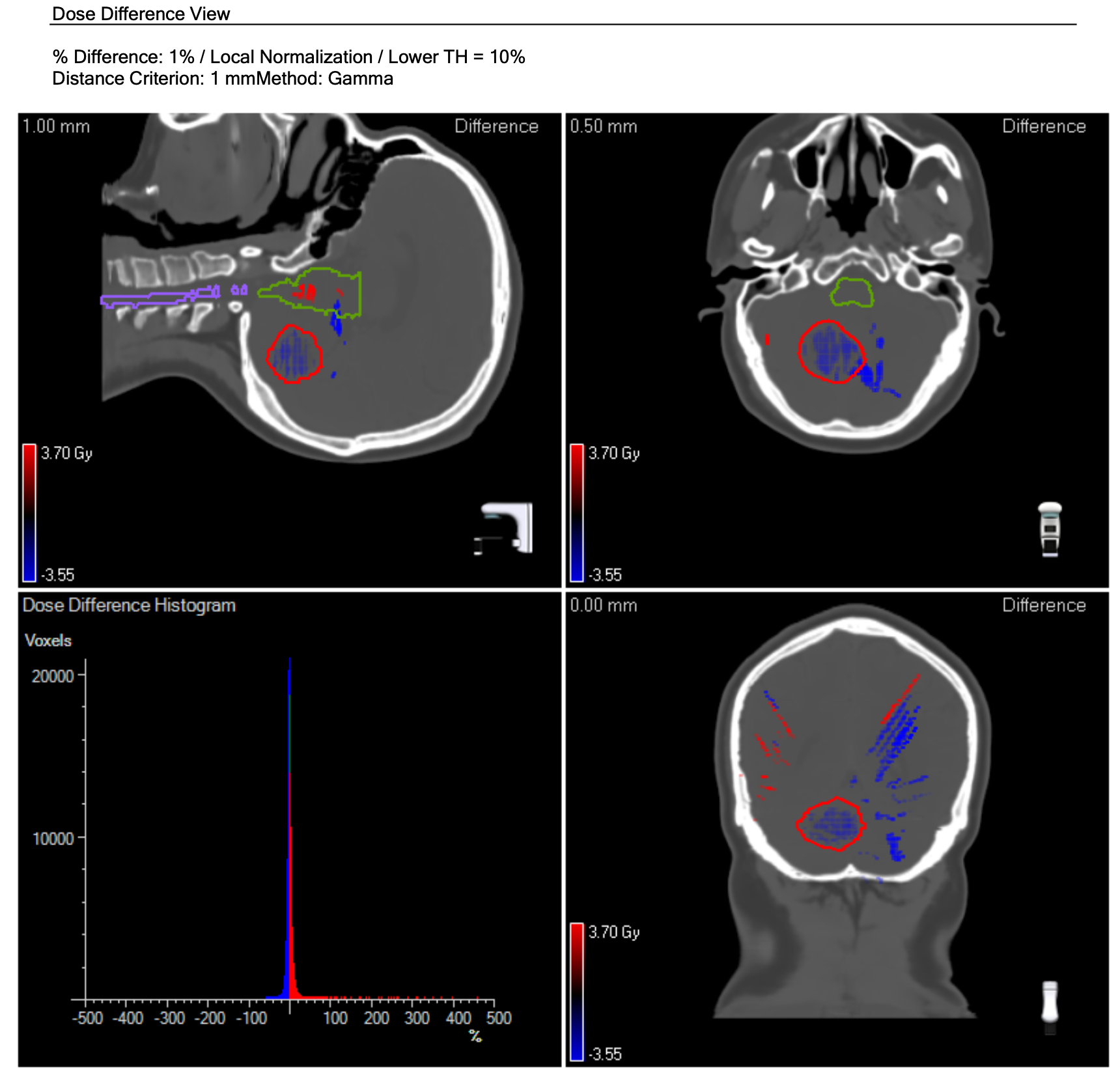


**Figure E1:** Example of dose comparison for 3DVH presentation of a patient with 3D gamma dose deviation calculation.


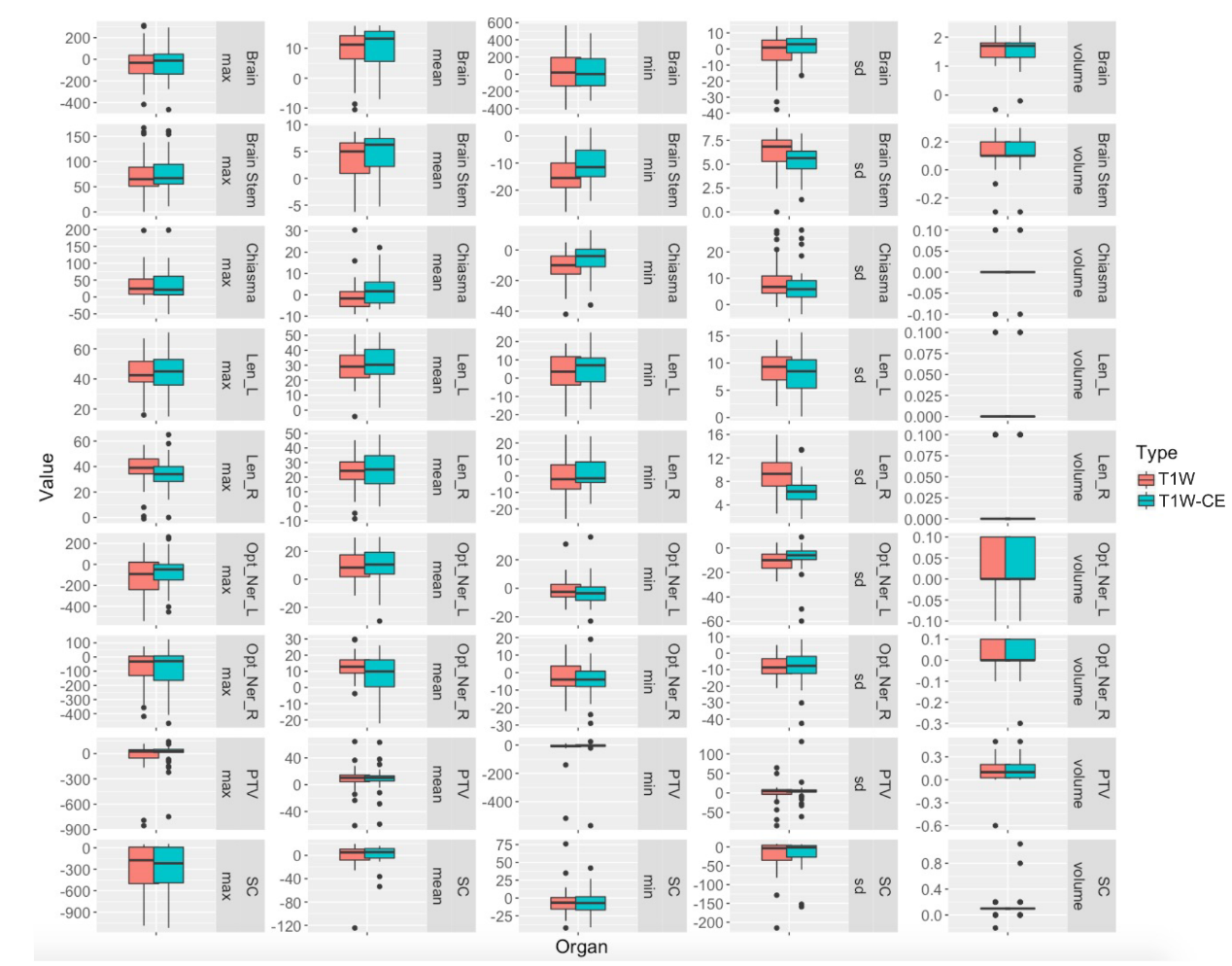


**Figure E2:** The box distribution of HU-max, HU-mean, HU-min, HU-Sd, and volume deviation values between planning CT and pseudo-CT (planning CT minus pseudo-CT).


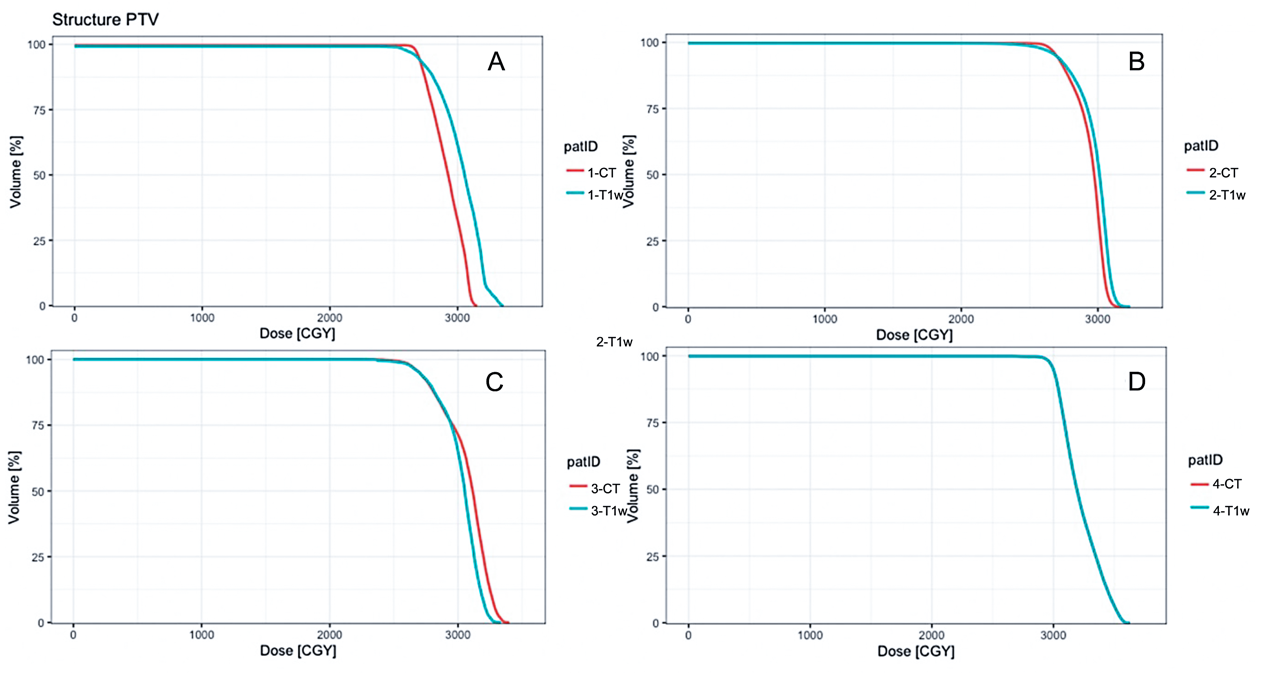


**Figure E3:** Some example diagrams of DVH. (A)-(B): 1%/1mm local gamma ≤ 80%, (C)-(D): 1%/1mm local gamma >80%.


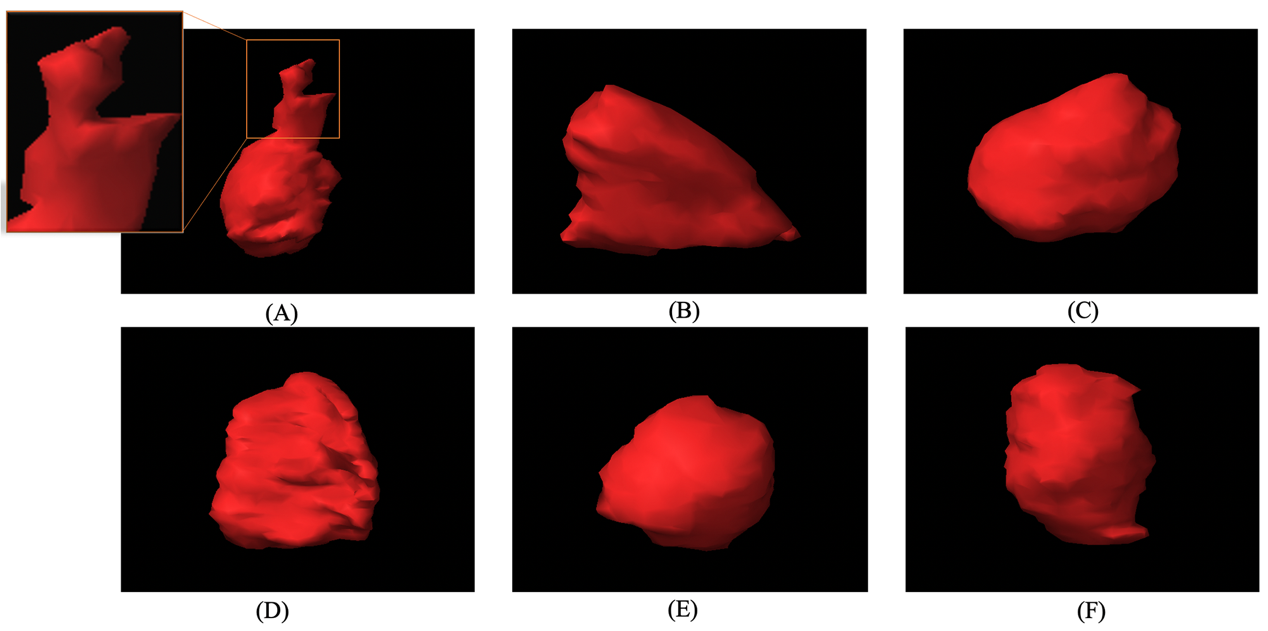


**Figure E4:** GTV’s morphology of all single lesions >10cc in 34 patients: (A) the only lesion with a 1%/1mm passage rate of ≤ 80%, (B)-(F) the remaining lesions with a pass rate of >80% in all.

**
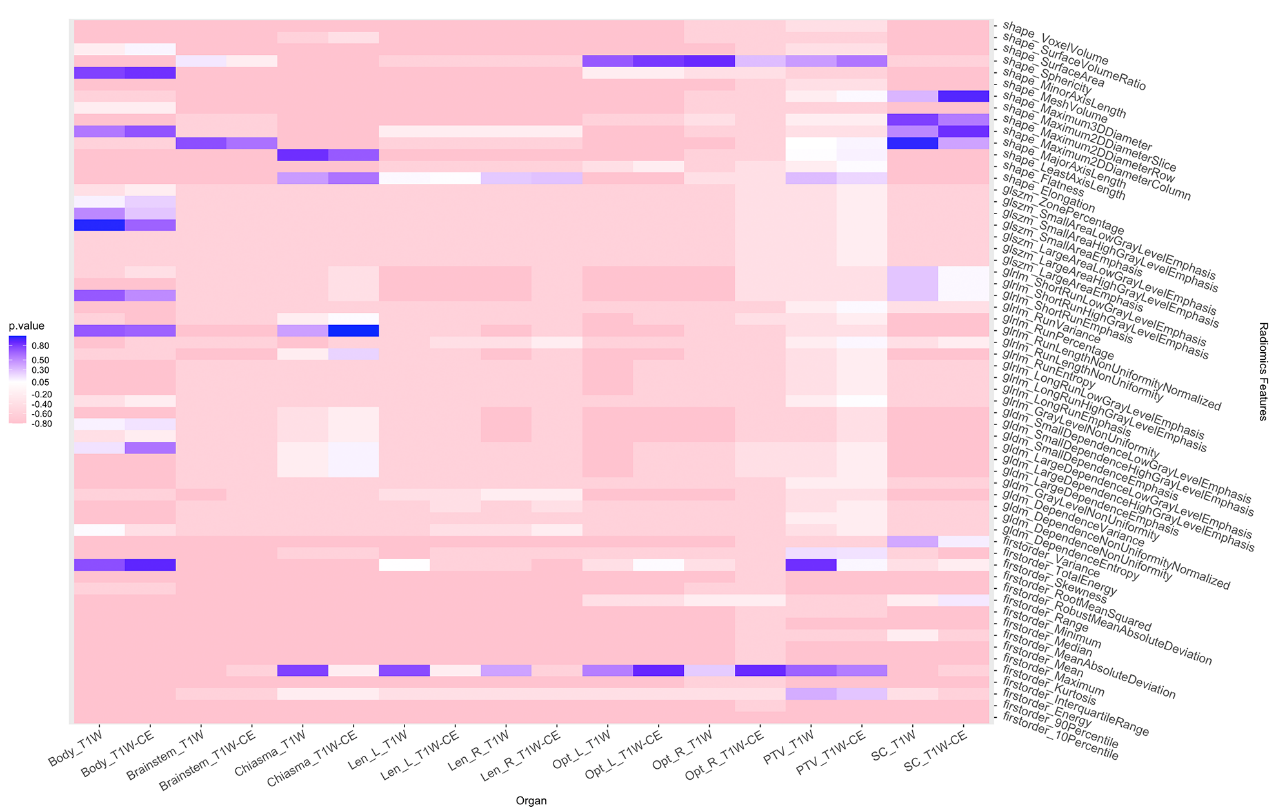
Figure E5:** The p-value heat map results for PTVs and OARs between the pseudo-CTs generated by T1w MRI and planning CTs.


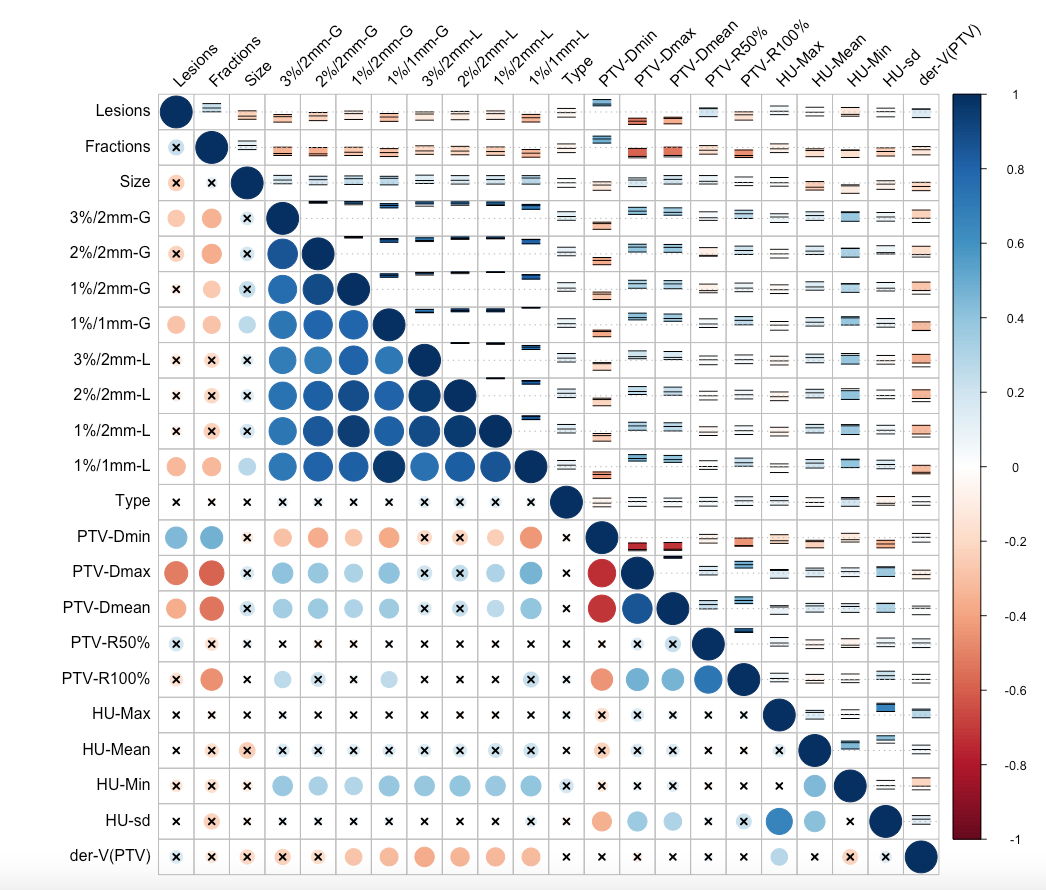


**Figure E6:** Confidence intervals for intra-group correlation.


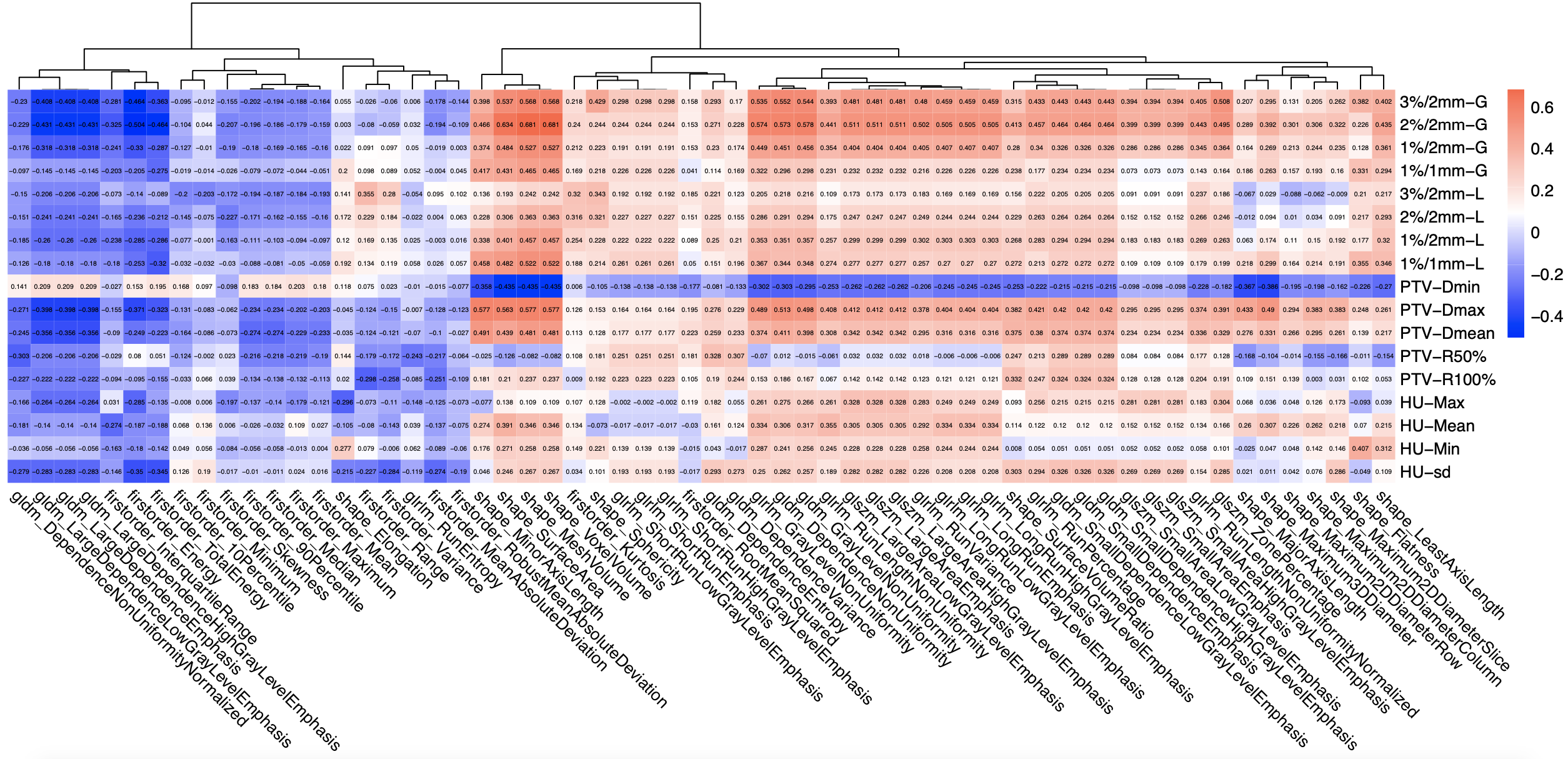


**Figure E7:** Values of inter-group correlation coefficients.
